# Supplementary material for: Accelerated Laboratory Weathering of Polypropylene/Poly (Lactic Acid) Blends
Source: Polymers (Basel). 2022 Dec 21;15(1):17. doi: 10.3390/polym15010017 (PMC9823939; doi:10.3390/polym15010017)
Supplement: Supplementary file 1 [file polymers-15-00017-s001.zip › polymers-2090914-supplementary.pdf]

Supporting Information

Accelerated Laboratory Weathering of Polypropylene/Poly (Lactic Acid) Blends

Qihua Zhou *, Xuan Liu, Yanzhen Lu, Xiaoyao Dao and Liuqing Qiu

School of Chemistry and Chemical Engineering, Hefei Normal University, Hefei 230601, China;
* Correspondence: zhouqihua@hfnu.edu.cn; Tel.: +86-551-63674145

Tensile and flexural tests were conducted with a CMT6104 microcomputer controlled electronic universal testing machine (Shenzhen Xinsansi Material Testing Co., Ltd, Shenzhen, China) according to ASTM D638 standard, the tensile test speed was fixed at 50 mm/min. Notched Impact testing was conducted according to ASTM D256 with a ZBC8400-B pendulum impact testing machine (Shenzhen Xinsansi Material Testing Co., Ltd, Shenzhen, China). In all cases, 5 specimens were tested, and the average values were reported. The PP / PLA8.2 and PP / PLA8.2c before weathering were quenched by liquid nitrogen, and the cross sections were sprayed with gold by ion sputtering instrument. The section morphology of PP/PLA blends was performed using scanning electron microscopy (SEM) observations (SU-1510, Hitachi, Tokyo, Japan) with an accelerating voltage of 10.0 kV.

Table S1. Characteristics of PP/PLA blends different compositions before weathering.

Blends	Tensile strength (MPa)	Elongation at break (%)	Flexural srength (MPa)	Flexural modulus (MPa)	Impact strength (kJ/m3)	ϱ(g/cm³)
PP/PLA10.0	24.46	72.48	21.48	923	3.64	0.90
PP/PLA9.1	22.7	71.92	26.15	1083	3.69	0.922
PP/PLA8.2	29.15	13.00	31.27	1281	4.57	0.949
PP/PLA7.3	29.15	7.35	37.09	1538	6.08	0.981
PP/PLA6.4	29.03	6.60	42.16	1669	6.19	1.008
PP/PLA5.5	29.42	6.00	47.05	1867	5.44	1.037

PP/PLA4.6	33.42	5.49	53.36	2200	5.45	1.069
PP/PLA3.7	37.96	5.77	56.01	2328	5.83	1.115
PP/PLA2.8	44.65	5.86	66.84	2618	5.20	1.153
PP/PLA1.9	52.18	6.60	73.17	2991	6.20	1.191
PP/PLA0.10	70.04	5.63	99.41	3663	3.96	1.280
PP/PLAs[1]	20.95	202.8	18.92	805	4.16	0.950

[1] PP/PLA sample provided by Jieshou Tianlu Packaging Materials Co., Ltd.

Table S2. Characteristics of PP/PLA8.2 and PP/PLA8.2c before weathering.

Blends	Tensile strength (MPa)	Elongation at break (%)	Flexural srength (MPa)	Flexural modulus (MPa)	Impact strength (kJ/m³)	ϱ(g/cm³)
PP/PLA8.2	29.15	13	31.27	1281	4.57	0.949
PP/PLA8.2c	23.78	33.86	27.01	1078	7.82	0.943

Table S3. T_i of blends before and after accelerated weathering 60 days.

Blends	Before accelerated weathering(℃)	After 60 days(℃)
PP/PLA10.0	412.8	344.0
PP/PLA9.1	329.7	324.0
PP/PLA8.2	332.2	320.2
PP/PLA7.3	338.2	332.5
PP/PLA6.4	325.7	311.6
PP/PLA5.5	328.6	305.4
PP/PLA4.6	326.4	305.6
PP/PLA3.7	325.6	303.5
PP/PLA2.8	330.8	254.7
PP/PLA1.9	333.3	299.8
PP/PLA0.10	334.8	328.3

Table S4. T_i of PP/PLA8.2 and PP/PLA8.2c blends before and after different accelerated weathering times.

Blends	0 d	30 d	60 d	90 d	150 d
PP/PLA8.2	332.2	334.7	320.2	274.8	291.3
PP/PLA8.2c	339.8	339.8	310.4	270.6	274.8

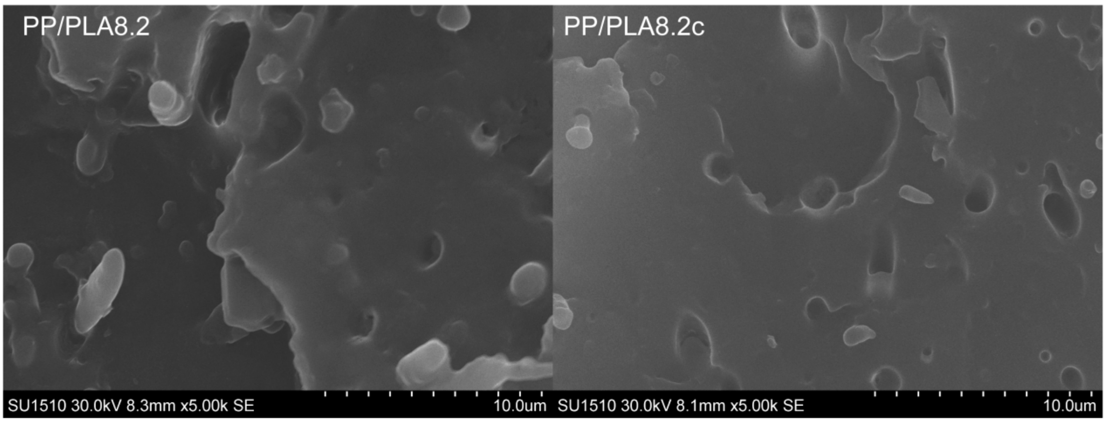

Figure S1. SEM of PP/PLA8.2 and PP/PLA8.2c blends before weathering.
